# Characterization and Biomedical Applications of Green-Synthesized Selenium Nanoparticles Using Tridax procumbens Stem Extract

**DOI:** 10.7759/cureus.63535

**Published:** 2024-06-30

**Authors:** Jayapriya Johnson, Rajeshkumar Shanmugam, Pradeep Manigandan

**Affiliations:** 1 Nanobiomedicine Lab, Centre for Global Health Research, Saveetha Medical College and Hospitals, Saveetha Institute of Medical and Technical Sciences, Chennai, IND

**Keywords:** scanning electron microscopy analysis, cytotoxic effect, anti-oxidant activity, anti-inflammatory effect, biomedical applications, antimicrobial activity

## Abstract

Background

Selenium nanoparticles (SeNPs) are one of the metal nanoparticles that have been widely utilized for their anti-microbial, anti-oxidant, anti-inflammatory activities, and other biomedical applications. *Tridax procumbens* (TP) stem extract is a promising herb species rich in flavonoids, tannins, alkaloids, phytosterols, and hydroxycinnamates, which play a major role in wound healing applications.

Aim

The study aims to synthesize SeNPs using TP stem extract*, *characterizations, and its biomedical applications.

Materials and methods

SeNPs were synthesized using TP stem extract. The green synthesis of SeNPs was confirmed by ultraviolet-visible (UV-vis) spectra analysis. The synthesized SeNPs were characterized using Fourier-transform infrared spectroscopy (FTIR) and scanning electron microscopy (SEM). The agar well diffusion method was utilized to evaluate the anti-bacterial properties of the green synthesized SeNPs using TP stem extract. The anti-oxidant effect of SeNPs was tested using the 2,2-diphenyl-1-picrylhydrazyl (DPPH) assay, ferric-reducing anti-oxidant power assay (FRAP), and hydroxyl radical scavenging assay (H₂O₂). The anti-inflammatory effect was investigated using the bovine serum albumin assay and egg albumin denaturation method, and the cytotoxic effect of the green synthesized SeNPs was tested using the brine shrimp lethality (BSL) assay.

Results

The green synthesis of SeNPs was confirmed using different types of analysis techniques. The characterizations were done by UV-visible spectroscopy analysis, exhibiting a maximum peak at the range of 330 nm. SEM analysis revealed the shape of the nanoparticle to be hexagonal. The agar well diffusion method exhibited the anti-bacterial efficacy of SeNPs against wound microorganisms with a zone of inhibition of 14.6 mm for *Escherichia coli* (*E. coli*), 15.8 mm for *Staphylococcus aureus* (*S. aureus*), and 15.4 mm for *Pseudomonas aeruginosa* (*P. aeruginosa*). The TP stem-mediated SeNPs showed potential effects in anti-oxidant, anti-inflammatory, and cytotoxic activity, which shows very little toxicity.

Conclusion

Overall, the green synthesis of TP-stem-mediated SeNPs has great potential in biomedical applications. Thus, the synthesized SeNPs exhibit significant anti-bacterial efficacy against wound pathogens. The TP stem-mediated SeNPs showed potential effects in anti-oxidant, anti-inflammatory, and cytotoxic activity, which shows low toxicity. Furthermore, the green-synthesized SeNPs can be utilized in therapeutic management.

## Introduction

Damage to the body's tissues, whether from accidents, surgeries, or long-term illnesses, is represented by wounds. In addition to their external appearance, wounds are susceptible to pathogen colonization, including bacteria, fungi, viruses, and other microorganisms. These microbes, aside from delaying the body's normal healing process, can cause substantial problems that negatively influence patient outcomes. Healthcare professionals must have a thorough understanding of wound pathogen dynamics and consequences to make informed judgments about infection prevention techniques, anti-microbial treatments, and wound management procedures. The context for this introduction's dive into the complex interactions between infections and wounds is established, highlighting the necessity of practical approaches that minimize the adverse effects of these interactions and encourage the most effective healing [1]. Furthermore, nanotechnology plays a vital role in wound healing and other biomedical applications. Nanotechnology refers to disciplines of science and engineering in which nanoscale phenomena are employed in the design, characterization, manufacturing, and applications of materials, structures, devices, and systems. Nanotechnology focuses on particles with dimensions smaller than 100 nm [2]. Nanotechnology is a multidisciplinary scientific discipline that is rapidly expanding, especially in biomedical applications. The development and utilization of materials with nanoscale dimensions in many aspects of life is the focus of nanotechnology. Because of their nanoscale size, nanoparticles exhibit highly specialized characteristics due to their high surface-to-volume ratios [3]. Nanotechnology has been quite effective in improving targeted drug delivery, as well as imaging, intracellular targeting, and therapeutic gene release, where most metal nanoparticles show significant impact [4].

Metal nanoparticles made up of Ag, Au, Ce, Fe, Se, Si, Ti, and Zn hold a distinctive place in the field of nanotechnology since they provide a unique possibility of serving not only as therapeutic agents but also as transporters for chemotherapeutic drugs, proteins, siRNA, and other molecules. Selenium (Se) is a crucial element for human health and may be obtained in nature from several sources [5]. Plants convert inorganic selenium from soil into selenomethionine using methionine as a protein precursor, resulting in dietary selenium. Selenium has an important role in several physiological processes, including proper growth, development, and anti-oxidant defense in fish, where its effects are achieved by the production of selenoproteins. Selenium nanoparticles (SeNPs) are more chemically stable, less toxic, and have great potential to slowly release Se ions after consumption, increasing the efficiency of selenoproteins production in the body [6,7]. A vast number of studies have shown that Se has a role in inducing cancer cell death. Electrochemical synthesis of SeNPs involves electrolysis of a fluid solution containing selenium dioxide. SeNPs are synthesized using microorganisms and a variety of plants using the green synthesis method because of their eco-friendliness and cost-effectiveness. According to recent findings, SeNPs have been researched for great impact in treating diabetes, Alzheimer's disease (AD), and inflammation-related disorders such as rheumatoid arthritis [8]. Although SeNPs are fundamentally inorganic selenium, their tiny size gives them unique chemical and physical characteristics such as strong antibacterial properties, low toxicity, and high biocompatibility. Owing to their elevated level of bioactivity, they are utilized in diverse biological applications. Green synthesis of nanoparticles using various plants creates a high impact on biomedical applications for treating a variety of diseases [9].

*Tridax procumbens* (TP) stem extract and leaves are used as both raw feed for cattle and a food additive for humans. The leaves have therapeutic properties and can cure diarrhea, gastroenteritis, and vomiting. Leaf and stem extracts are used as antiseptics for treating cuts, wounds, and burns. It also has hair growth-boosting properties [10]. TP stem is a very promising species that generates secondary metabolites with a wide range of medical applications, including anti-anemic, anti-inflammatory, anti-diabetic, and anesthetic effects. This plant has a lengthy history of traditional usage among several cultures. The biological evaluation included tests for anti-bacterial, anti-cancer, anti-fertility, anti-fungal, anti-helminthic, anti-protozoal, anti-viral, and other pharmacological activities. The aqueous extract of TP leaves containing essential oils proved anti-metastatic activity against lung cancer development in C57BL/6 (B16 F-10 melanoma cell line) mice [11].

In this study, SeNPs were synthesized using TP stem extract. The green-synthesized SeNPs were analyzed using ultraviolet-visible (UV-vis) spectroscopy, Fourier-transform infrared spectroscopy (FTIR), and scanning electron microscope (SEM). The anti-microbial property of the synthesized SeNPs was assessed using the agar well diffusion method. To investigate its anti-oxidant effect on 2,2-diphenyl-1-picrylhydrazyl (DPPH), ferric reducing anti-oxidant power (FRAP) assay, and hydrogen peroxide scavenging (H₂O₂) assay, were utilized. The anti-inflammatory effect was evaluated using an egg albumin (EA) denaturation and bovine serum albumin (BSA) assay, and the cytotoxic effect was tested using a brine shrimp lethality (BSL) assay.

## Materials and methods

Collection of plant samples

The stem of TP was collected and washed with double distilled water repeatedly to remove debris. Then, the plants were shade-dried at ambient temperature. Next, the stem parts were dried and powdered. Two grams of TP stem were weighed and added to a conical flask containing 100 mL of double-deionized water. The solution was boiled for 15-20 minutes using a heating mantle set at a temperature of 60°C. After boiling, the solution was filtered using grade No. 1 Whatman filter paper. The filtered aqueous extract is used for further studies.

Green synthesis of SeNPs

A 20 mM selenium solution was prepared by adding 0.346 g of sodium selenite to 70 mL of distilled water, to which 30 mL of freshly prepared TP stem extract was added. The mixture was retained on an orbital shaker for 48-72 hours. The synthesized TP stem-mediated SeNP pellets were obtained through centrifugation at 10,000 rpm (revolutions per minute) for 10 minutes. The supernatant was discarded, and the pellet was collected, dried at 70°C, and then stored in an air-tight container. The preserved pellets were further utilized for characterization purposes.

Characterization techniques

The expansion of technology has led us to explore and evolve various characterization techniques, especially in nanoparticle characterization. Periodic sampling of the aliquots of the green-synthesized SeNPs was monitored using a double-beam UV-vis spectrophotometer (ESICO - Model 3375, ESICO Triton International, Brookfield, USA). The maximum absorbance was recorded within the wavelength range from 300 to 650 nm. The functional groups of SeNPs can be identified using FTIR with wavelengths ranging from 500 to 3500 cm^-1^. The surface morphology was characterized using SEM.

Anti-bacterial activity

The anti-bacterial activity of TP stem-mediated SeNPs was tested against wound pathogens such as *Escherichia coli *(*E. coli*), *Pseudomonas aeruginosa *(*P. aeruginosa*), and *Staphylococcus aureus *(*S. aureus*). Mueller Hinton Agar (MHA) was sterilized for 15 minutes at 121°C. Media was poured into the Petri plates and allowed to solidify. Wells were cut on the agar plate, and each organism was swabbed, respectively. The nanoparticles with various concentrations (25, 50, 100 µg/mL) were loaded. Mupirocin was used as an antibiotic in this activity, and the agar plates were incubated for 24 hours at 37°C. After the incubation period, the zone of inhibition observed around the well was enumerated.

Anti-oxidant activity

DPPH Assay

The DPPH assay was utilized to examine the anti-oxidant level for different concentrations (10-50 μg/mL) of TP stem-mediated SeNPs. The assay was carried out by using TP stem-mediated SeNPs dissolved in 1 mL of 0.1 mM DPPH in methanol. Then, 450 µL of 50 mM Tris-HCl was added and maintained at pH 7.4 for half an hour. The reduction in the amount of DPPH free radicals at different concentrations was monitored at 517 nm. Butylated hydroxytoluene was used as a control [12]. The percentage of inhibition was calculated using the formula below:

$\text{Percentage of inhibition} = \frac{\text{Absorbance of control} - \text{Absorbance of sample}}{\text{Absorbance of control}} \times 100$

FRAP Assay

A FRAP assay was carried out to identify the antioxidants present in the TP stem-mediated SeNPs and the amount it reduced from Fe^+3^ to Fe^+2^. Firstly, a FRAP solution of 3.6 mL was taken. Then, 0.4 mL of distilled water was added. Followed by the addition of TP stem-mediated SeNPs (10-50 µg/mL), the complete mixture was maintained in a dark room for 10 minutes. The absorbance was measured at 593 nm for five concentrations of FeSO_4_·7H_2_O of TP stem-mediated SeNPs in FRAP reagent [13]. The percentage of inhibition was calculated using the formula below:

$\text{Percentage of inhibition} = \frac{\text{Absorbance of control} - \text{Absorbance of sample}}{\text{Absorbance of control}} \times 100$

Hydroxyl Radical Scavenging Assay

The hydroxyl radical scavenging assay was performed according to the Halliwell method. The reaction mixture contained 100 µL of 28 mM 2-deoxy-2-ribose (pH 7.4), 200 µL of FeCl_3_, followed by 1.04 mM of EDTA, 1.0 mM of H_2_O_2_, and 100 µL of ascorbic acid. Different concentrations of TP stem-mediated SeNPs (10-50 µg/mL) were added to the reaction mixture. The mixture was left undisturbed for an hour at room temperature. After the thiobarbituric acid reaction, absorbance was measured at 532 nm using a UV spectrophotometer, with ascorbic acid as the standard and vitamin E as the control [14]. The percentage of inhibition was calculated using the formula below:

$\text{Percentage of inhibition} = \frac{\text{Absorbance of control} - \text{Absorbance of sample}}{\text{Absorbance of control}} \times 100$

Anti-inflammatory effect

BSA Assay

The mechanism of anti-inflammatory activity was assessed by proteinase enzyme inhibitory action. Firstly, 0.45 mL of BSA was introduced, and then 1N HCl was added to maintain pH 6.3. After that, TP stem-mediated SeNPs were added at different concentrations (10-50 µg/mL). The reaction mixture was maintained at room temperature for 20 minutes, followed by exposure to a water bath for 30 minutes at 55°C. Finally, it was cooled and measured at 660 nm [15]. The percentage of inhibition was calculated using the formula below:

$\text{Percentage of inhibition} = \frac{\text{Absorbance of control} - \text{Absorbance of sample}}{\text{Absorbance of control}} \times 100$

Egg Albumin Denaturation Assay

The egg albumin denaturation assay was set up with 2.8 mL of phosphate-buffered saline maintained at pH 6.4 and 0.2 mL of (hen’s) egg albumin. Thereafter, 10, 20, 30, 40, and 50 µg/mL of TP stem-mediated SeNPs were added to the mixture. The TP stem-mediated SeNPs, along with the reaction mixture, were kept at room temperature for 10 minutes. After cooling, their absorbance was measured at 660 nm. Distilled water was used as a negative control, and diclofenac was employed as a positive control [16]. The percentage of inhibition was calculated using the formula below:

$\text{Percentage of inhibition} = \frac{\text{Absorbance of control} - \text{Absorbance of sample}}{\text{Absorbance of control}} \times 100$

BSL Assay

To facilitate brine shrimp hatching, 36 g of sea salt was dissolved in 1 L of distilled water to create artificial seawater. The seawater was placed in a tiny plastic vessel with a division separating the light and dark (covered) areas (the hatching chamber). Brine shrimps were placed in the chamber's dark side, and newly hatched shrimp were drawn to the lamp above the other side of the chamber (which emits regular light). The eggs were given one day to develop into brine shrimp (nauplii). After 24 hours, 10 brine shrimps were placed in vials accommodating 5, 10, 20, 40, and 80 µL of TP stem-mediated SeNPs. One well with 10 brine shrimps was kept as a control, and nanoparticles were not added. The number of live brine shrimp nauplii after 24 hours was counted manually by observing under a dissection microscope, and results were noted [17]. The percentage of live nauplii was calculated using the formula below:

$\textit{Percentage of live nauplii} = \frac{\textit{No of live nauplii} - \textit{No of dead nauplii}}{\textit{No of dead nauplii}} \times 100$

Statistical analysis

All experiments in this study were expressed as mean values ± standard deviation (SD) of triplicate measurements (n = 3). Significant differences (p < 0.05) were observed in the in vitro studies of anti-bacterial, anti-oxidant, anti-inflammatory, and cytotoxic activities. A one-way ANOVA test was used among treatment mean groups; IBM SPSS Statistics for Windows, Version 23 (Released 2015; IBM Corp., Armonk, NY, USA) was used for data processing.

## Results

Visual observation

**Figure 1:**
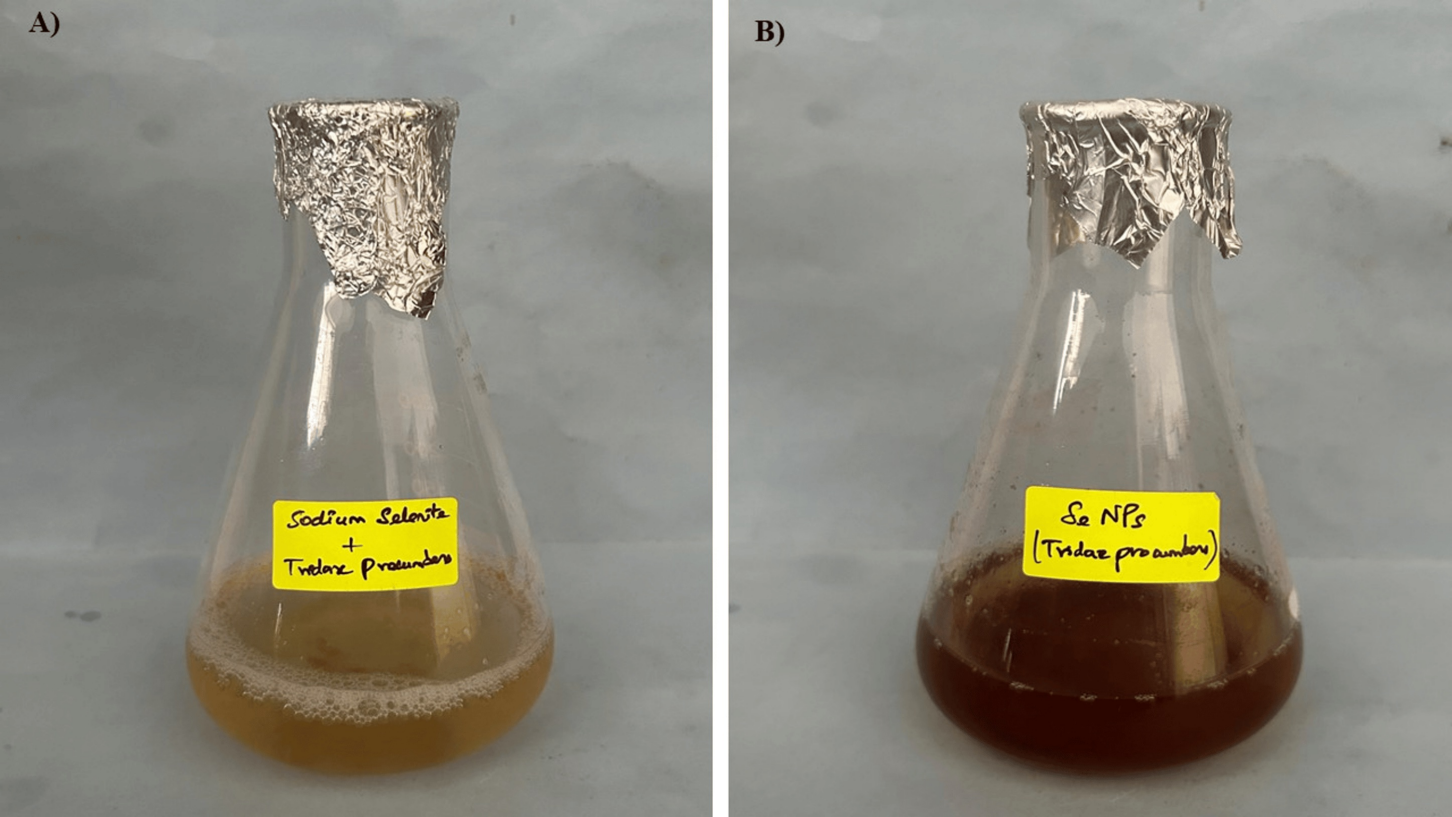
Green synthesis of SeNPs using TP stem extract A) Before incubation; B) After 48 hours of incubation SeNPs: Selenium nanoparticles; TP: *Tridax procumbens*

The reduction of the metal ions into metal nanoparticles incorporated by the plant extract has led to the color change of the solution. This shows the capping and reducing ability of the plant extract. In Figure 1, TP stem-mediated SeNPs gently changed their color from light yellow to a darker color after 48 hours.

UV-vis spectra analysis

**Figure 2:**
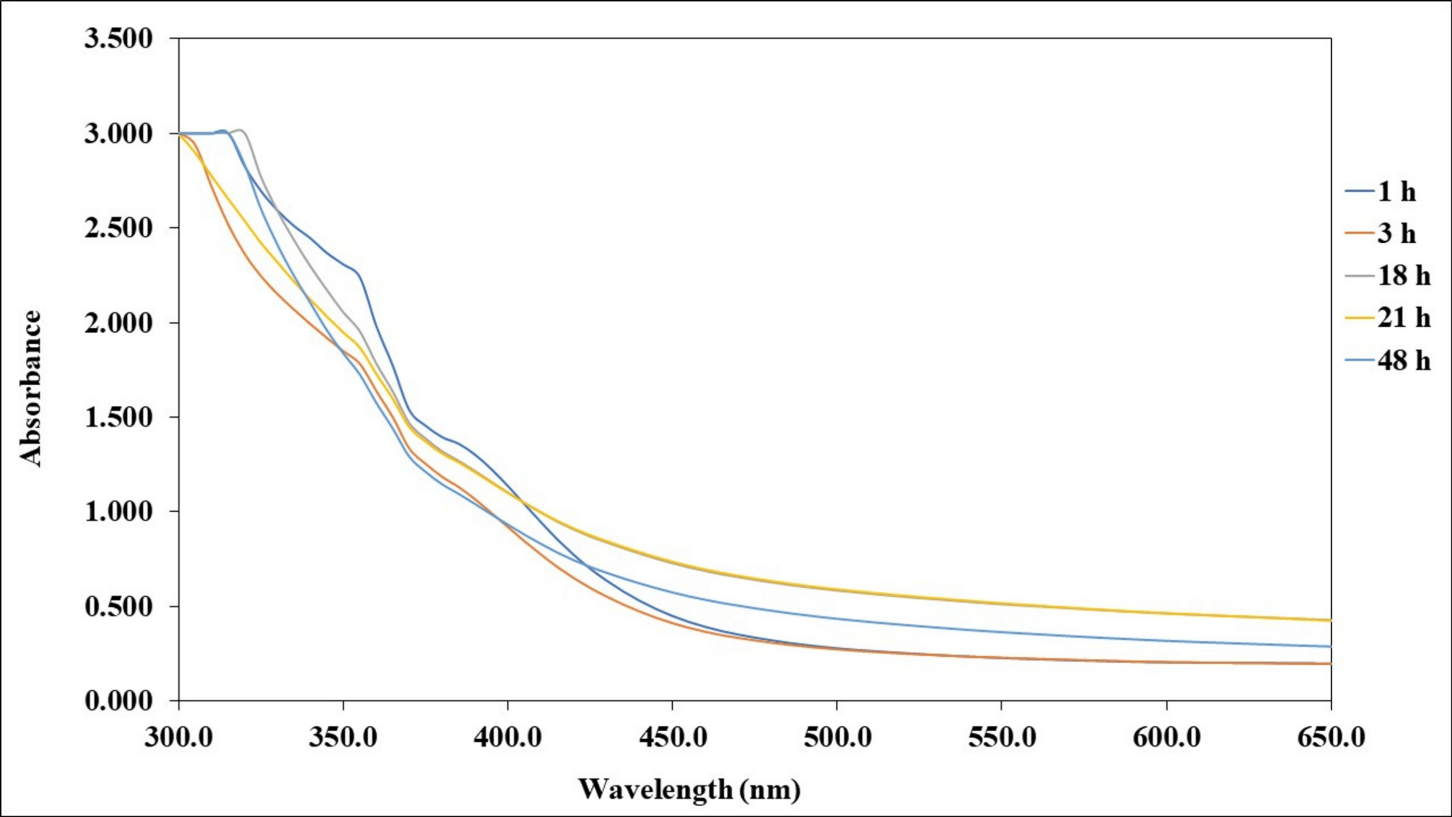
UV-vis spectra analysis of green synthesized SeNPs under different interval times SeNPs: Selenium nanoparticles

The synthesis of SeNPs by the reducing agent TP stem extract was preliminarily confirmed by a UV-vis spectrophotometer in the wavelength range of 300-650 nm. The spectra analysis was documented at (1, 3, 18, 21 and 48 hours) interruption. As shown in Figure 2, the UV spectra of SeNPs showed its absorption peak at 330 nm.

FTIR analysis

**Figure 3:**
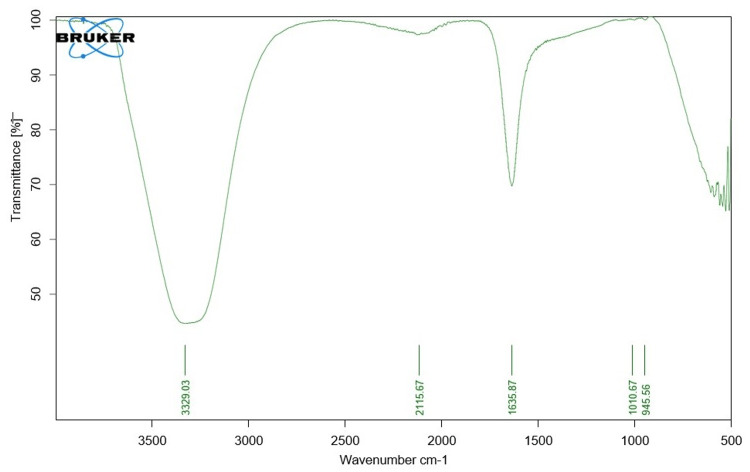
Fourier-transform infrared spectroscopy analysis of TP stem-mediated SeNPs SeNPs: Selenium nanoparticles; TP: *Tridax procumbens*

FTIR spectroscopy analysis was utilized in this study to identify the primary functional groups found in TP stem-mediated SeNPs. In Figure 3, the band at 3329.03 cm⁻¹ is ascribed to the stretching vibration of O-H groups in alcohols, phenols, and water. This is followed by a band at 2115.67 cm⁻¹, which appears due to the ≡C-H stretch in alkynes. The peak at 1635.87 cm⁻¹ is caused by the presence of alkenes (C=C symmetric stretch), aldehydes (C=O stretch), and amides (C=O stretch). Finally, a band at 1010 cm⁻¹ corresponds to the C-O stretch in esters and ethers. These results proved the presence of functional groups as biomolecules that are responsible for both the reduction and stabilization of SeNPs.

SEM

**Figure 4:**
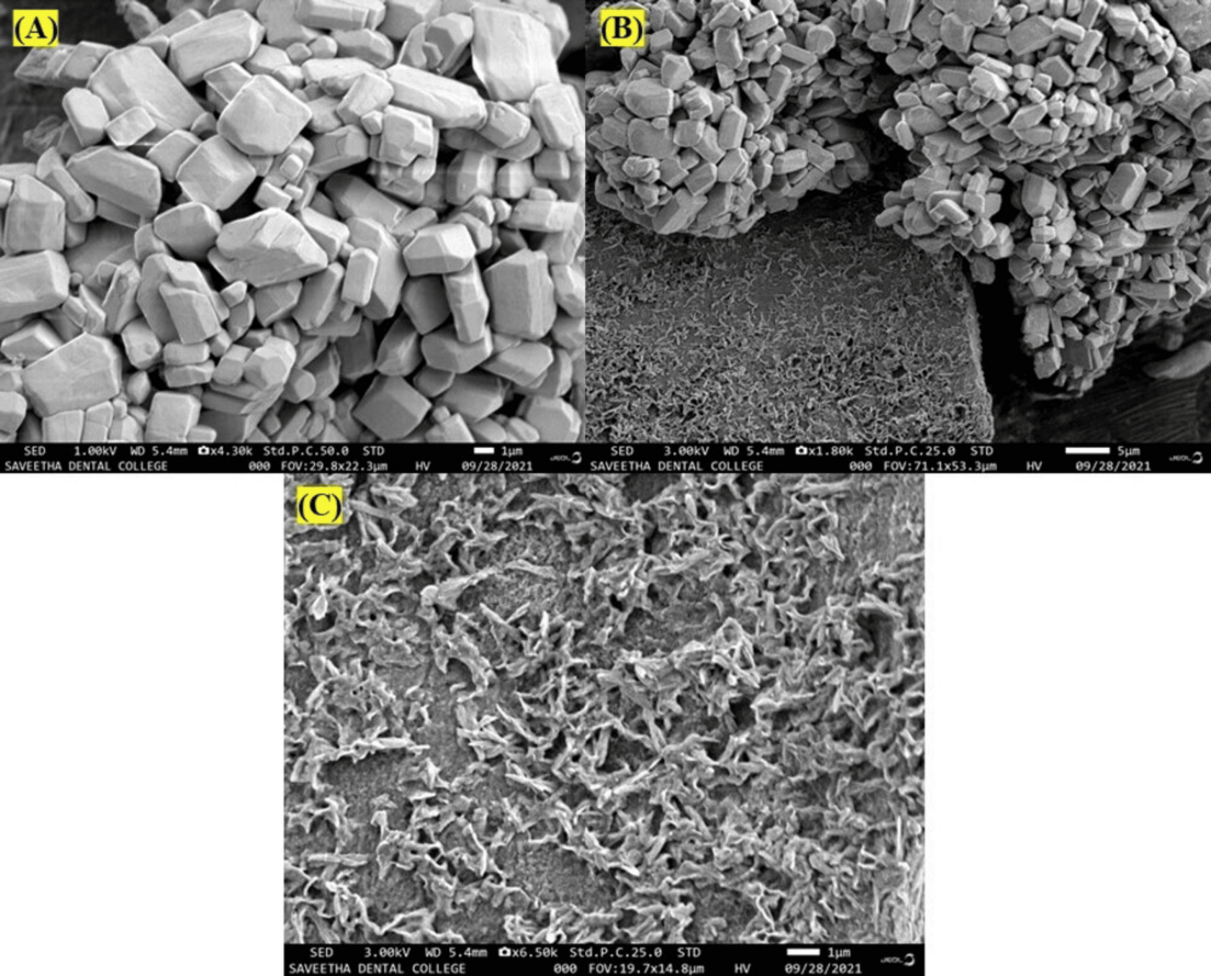
A)-C) Green synthesized SeNPs using TP stem under a scanning electron microscope SeNPs: Selenium nanoparticles; TP: *Tridax procumbens*

SEM is a kind of electron microscope that uses a high-energy electron beam scanned in a raster scan pattern to transfer the picture to the material. considering their numerous advantageous qualities, including excellent conductivity, chemical stability, and catalytic and anti-bacterial activity, smaller Se nanoparticles might find many advantageous applications. When the characteristics of the nanoparticles were tested using SEM. In Figure 4, the green synthesized SeNPs were found to be hexagonal and irregular in shape.

Anti-bacterial activity

**Figure 5:**
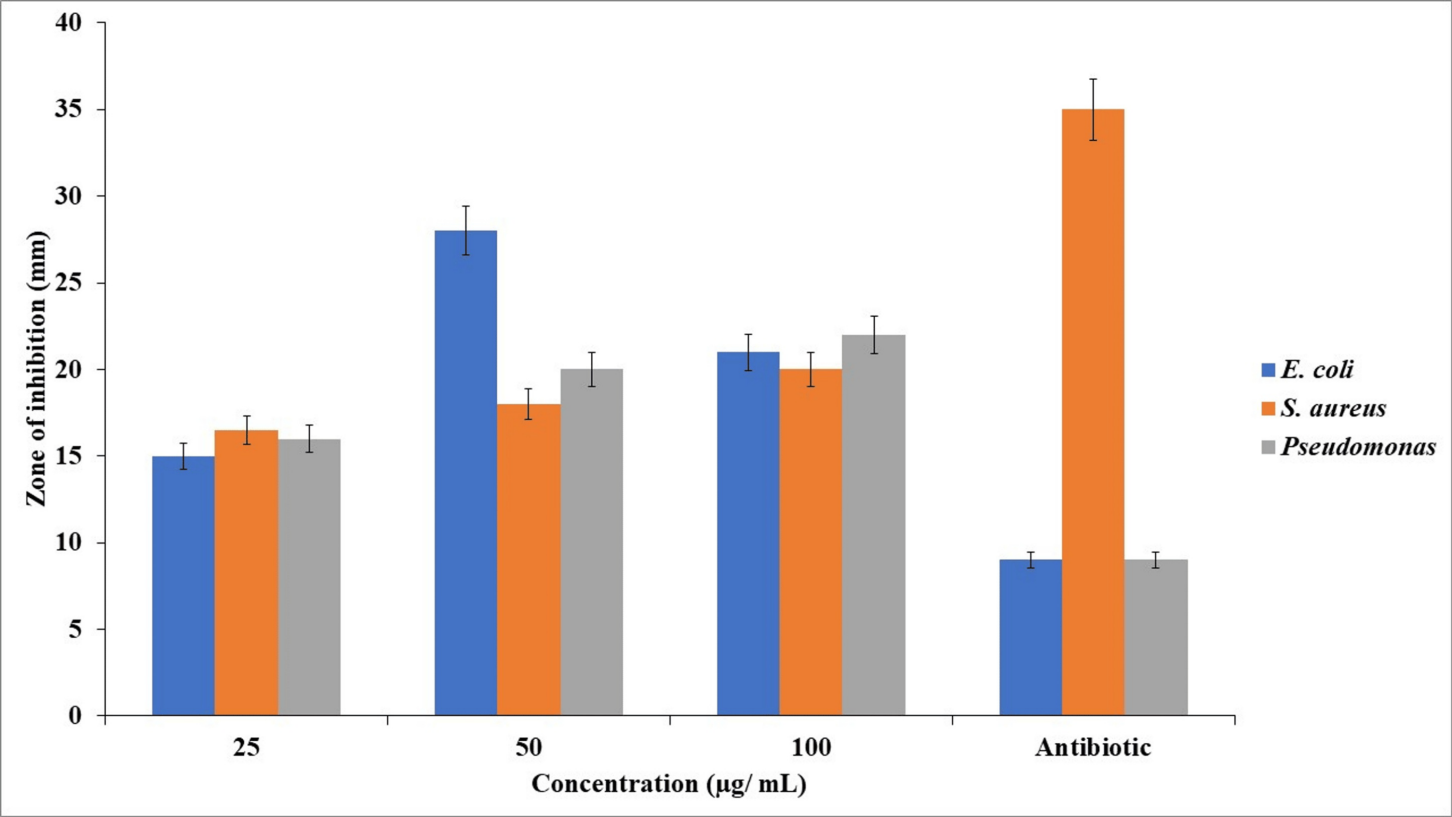
Antibacterial activity of TP stem-mediated SeNPs against wound pathogens TP: *Tridax procumbens*; SeNPs: Selenium nanoparticles

The agar well diffusion technique exhibits anti-microbial activity against different wound pathogens such as *P. aeruginosa*, *E. coli*, and *S. aureus*. TP stem-mediated SeNPs at a concentration of 25 µg/mL showed 14.6 ± 0.51% in *E. coli*, 15.83 ± 0.76% in *S. aureus*, and 15.4 ± 0.52% in *P. aeruginosa*. In the control group, it showed 8.4 ± 0.50%, 33.03 ± 1.00%, and 9.3 ± 0.57%, respectively. At a concentration of 50 µg/mL, *E. coli* showed 18.6 ± 0.6%, *S. aureus* showed 17.4 ± 0.56%, and *P. aeruginosa* showed 19.6 ± 0.52%. For the 100 µg/mL concentration, the inhibitory percentage for *E. coli* was 20.4 ± 0.50%, for *S. aureus* was 19.4 ± 0.51%, and for *P. aeruginosa* was 20.9 ± 1.67%, as shown in Figure 5.

Anti-oxidant effect

**Figure 6:**
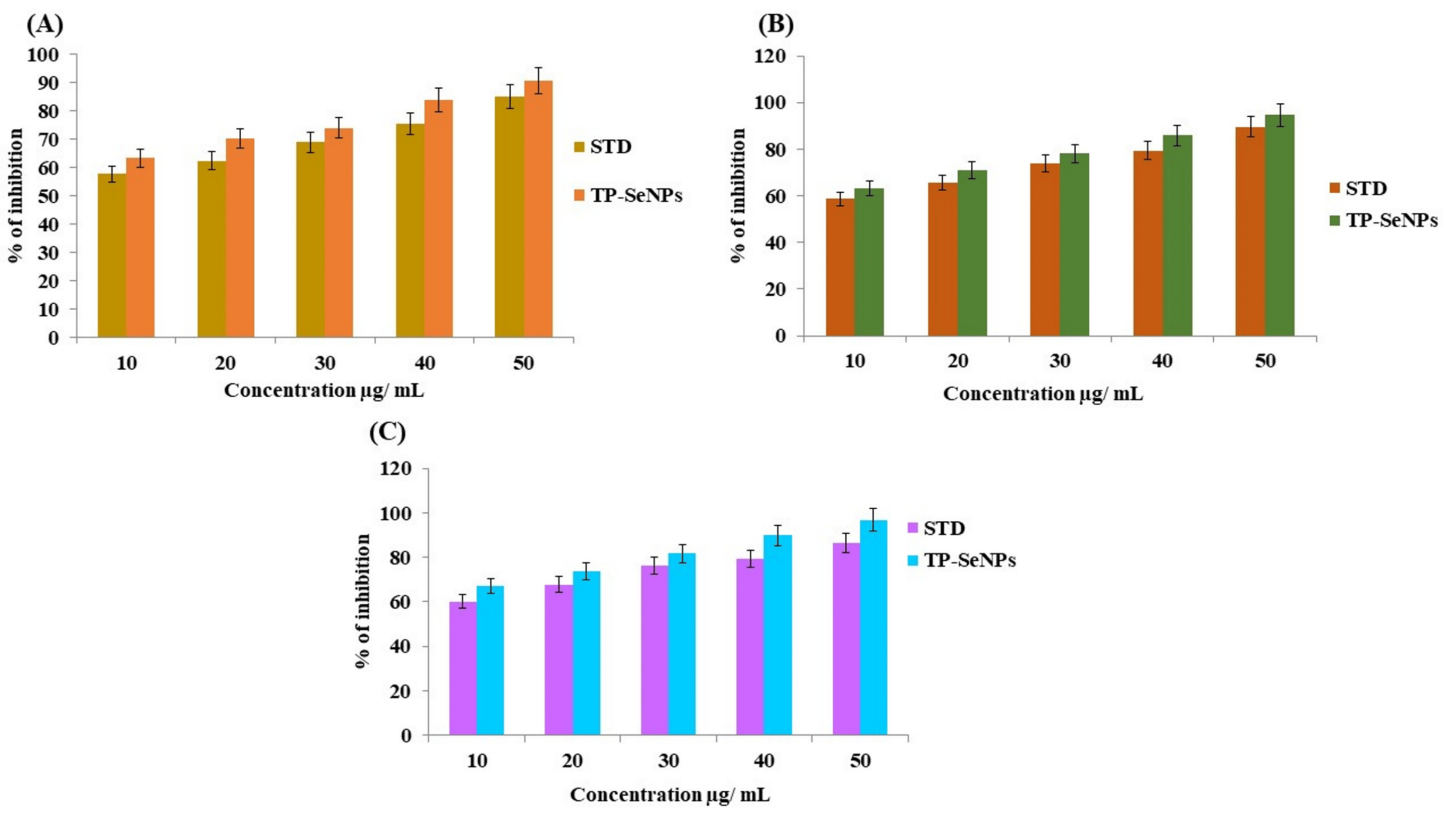
Anti-oxidant effect of TP stem-mediated SeNPs A) DPPH assay using TP stem-mediated SeNPs; B) Ferric reducing anti-oxidant power assay of green synthesized SeNPs using TP stem; C) Hydrogen peroxide radical free scavenging activity of TP stem-mediated SeNPS TP: Tridax procumbens; SeNPs: Selenium nanoparticles; DPPH: 2,2-diphenyl-1-picrylhydrazyl

TP stem-mediated SeNPs aqueous extract was tested for its anti-oxidant activity by DPPH, FRAP, and H_2_O_2_ methods. The scavenging rate analyzed by the DPPH method was expressed as a percentage. The scavenging rate increased with an increase in concentration (10-50 µg/mL). In Figure 6A, the 50 µg/mL maximum concentration revealed 90.5 ± 0.13%, while the same concentration for standard ascorbic acid showed 85.86 ± 0.39%. In the FRAP method, the free radical inhibition rate was higher at maximum concentration, with TP stem-mediated SeNPs showing 84.74 ± 0.01%, whereas the standard group showed 83.03 ± 0.03%, as shown in Figure 6B. In the H_2_O_2_ method, the maximum inhibition percentage at 50 µg/mL concentration was 89.54 ± 0.04%, while the standard group showed 83.54 ± 0.06%, as represented in Figure 6C.

Anti-inflammatory activity

**Figure 7:**
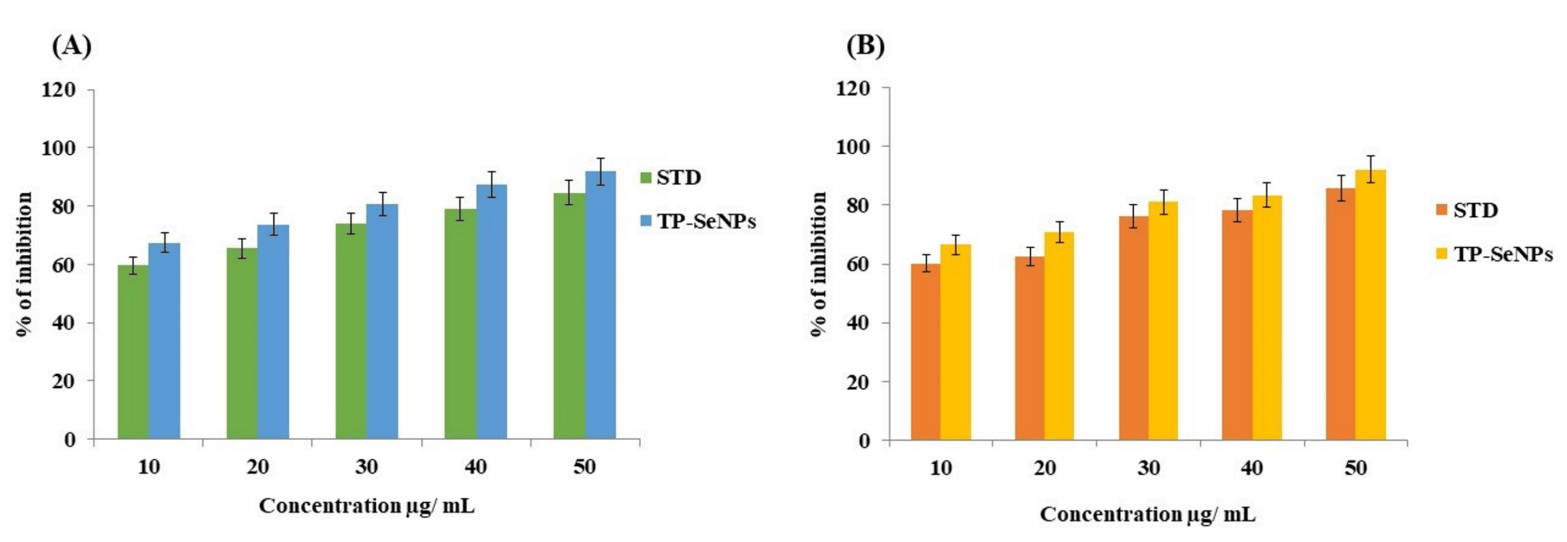
Anti-inflammatory activity of green synthesized using TP stem A) Egg albumin denaturation assay for synthesized SeNPs; B) Bovine serum albumin assay of TP stem-mediated SeNPs TP: Tridax procumbens; SeNPs: Selenium nanoparticles

The ability of the substance to inhibit the denaturation of proteins signifies the potential for anti-inflammatory activity. The TP stem-mediated SeNPs were analyzed for anti-inflammatory activities using BSA and EA assays. The mediated SeNPs were compared with diclofenac, which was used as the standard group. Both assays increased their inhibitory percentage with an increase in concentration from 10 to 50 µg/mL. In Figure 7A, the BSA activity of the synthesized SeNPs revealed 90.29 ± 0.74%, while the standard showed 83.3 ± 0.25% inhibition. In Figure 7B, the EA assay showed that the synthesized SeNPs had a maximum inhibitory percentage at 50 µg/mL, ranging from 85.48 ± 0.06%, while the standard showed 82.29 ± 0.71%. When compared to the standard group, TP stem-synthesized SeNPs proved to have greater inhibitory properties.

BSL assay

**Figure 8:**
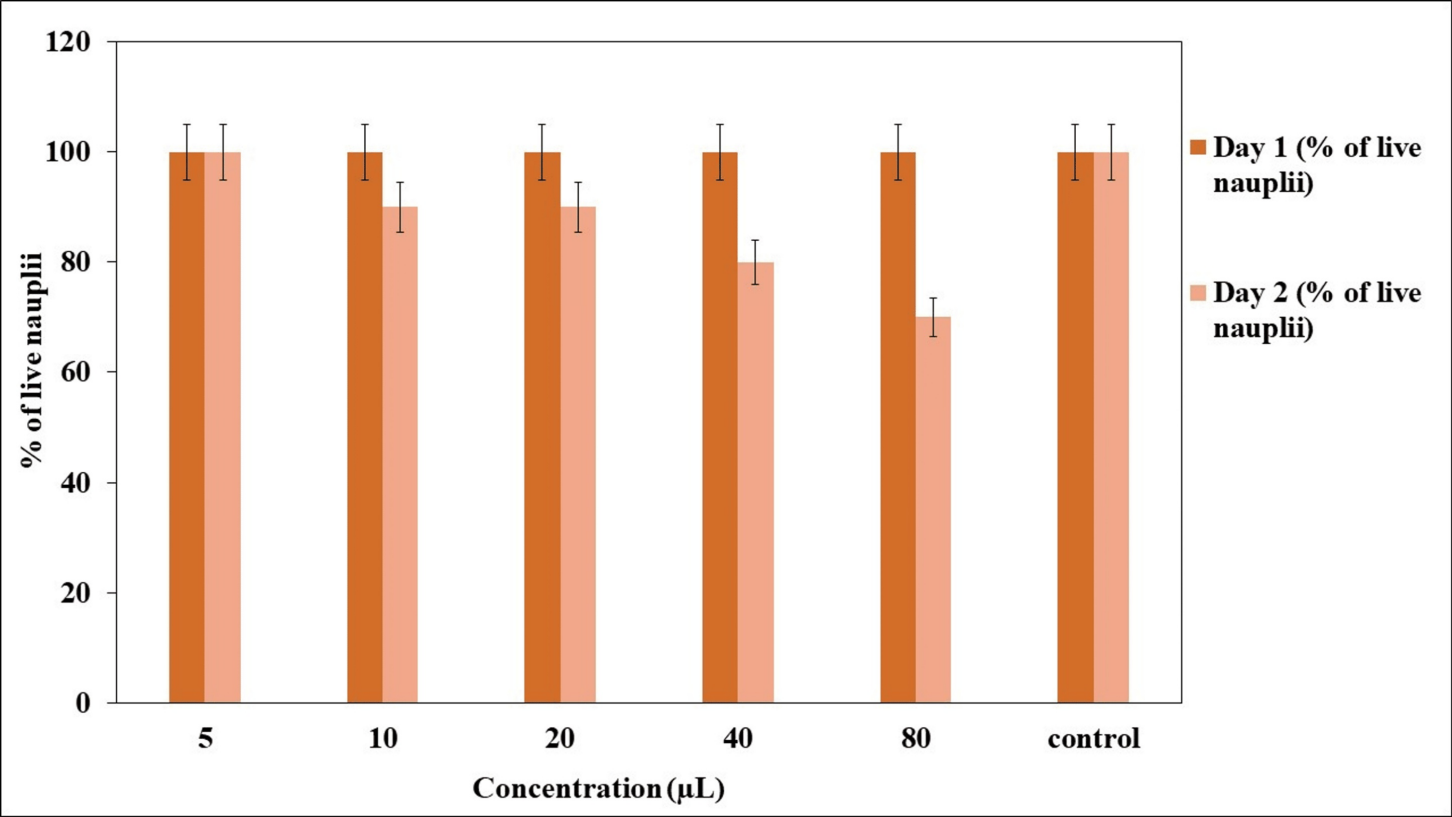
Cytotoxic effect of green synthesized SeNPs using brine shrimp lethality assay SeNPs: Selenium nanoparticles

The cytotoxicity effect of TP stem-mediated SeNPs was performed using the BSL assay. After 24 hours, there were 100% live nauplii present in the control group and the 5 µg/mL concentration group. In Figure 8, at the 10 µg/mL concentration, there was 90.62 ± 4.37% viability observed. Following this, there was 86.66 ± 3.54% viability at 20 µg/mL, 80 ± 2.46% live nauplii at 40 µg/mL, and 73.33 ± 6.76% viability at 80 µg/mL. The results exhibited that the TP stem-mediated SeNPs showed minimal toxicity, which can be used for further pharmaceutical applications.

## Discussion

The results presented in the study demonstrate the potential of TP stem extract in synthesizing SeNPs with significant biomedical applications. The synthesis process involves the reduction of metal ions into metal nanoparticles, which is facilitated by the plant extract. This process leads to a color change in the sample, indicating the capping and reducing the ability of the plant extract. The UV spectroscopy analysis confirms the synthesis of SeNPs, showing an absorption peak at 330 nm, which is characteristic of SeNPs. The synthesis of SeNPs using plant extracts has been a subject of interest in recent studies, with various plants demonstrating the ability to mediate the biosynthesis of SeNPs. For instance, a study on parched *Vitis vinifera* (raisins) showed that the presence of SeNPs was established by turning the extract from colorless to brick red, a change initiated by the mediated nano selenium particles along with the extract of the raisins [18]. This biological change was observed after five days of incubation, with the maximum absorbance at 270 nm for nano-selenium, indicating the successful synthesis of SeNPs. Another study focused on *Allium sativum* (garlic), which is considered a medicinally predominant plant, and found that the visual observation of the extract, initially colorless, turned to brick red after five days of incubation [19]. This study also reported a maximum absorbance at 270 nm for nano-selenium, similar to the findings with raisins, suggesting a consistent method for SeNP synthesis across different plant species. In contrast, a study on *Brassica oleracea* (cabbage) reported a peak at 420 nm for SeNPs, indicating a variation in the synthesis process or the specific conditions under which SeNPs are formed. This variation underscores the complexity of the biosynthesis process and the potential for different plant species to mediate the synthesis of SeNPs with varying properties [20,21].

SEM reveals that the synthesized SeNPs are hexagonal and have a size range of 30 to 150 nm, which is within the optimal size for various biomedical applications. In previous studies, the size and shape of the synthesized SeNPs have been consistently reported as spherical, with sizes ranging from 30 to 150 nm [22]. This uniformity in size and shape is crucial for the potential applications of SeNPs in biomedical fields, as these properties can influence their biocompatibility and therapeutic efficacy.

FTIR analysis further supports the synthesis of TP-mediated SeNPs by identifying the presence of functional groups such as O-H groups in alcohol, phenols, and water, as well as C-H stretch alkynes, C-C=C symmetric stretch, aldehydes, amides, and C-O stretch esters and ethers. These functional groups are crucial for the reduction and stabilization of SeNPs [23].

The biomedical applications of TP stem-mediated SeNPs are multifaceted, including anti-bacterial, anti-oxidant, anti-inflammatory, and cytotoxicity effects. The anti-bacterial activity is demonstrated against wound pathogens such as *P. aeruginosa*, *E. coli*, and *S. aureus*, with the SeNPs showing a higher inhibitory percentage compared to the control group. The anti-oxidant activity is evidenced by the DPPH, FRAP, and H_2_O_2_ methods, showing a scavenging rate that increases with concentration, reaching up to 90.5% at a 50 µg/mL concentration, which is comparable to the standard ascorbic acid. The anti-inflammatory effect is demonstrated through BSA and EA assays, showing an increase in inflammatory percentage with concentration, with the synthesized SeNPs exhibiting greater inhibitory properties compared to the standard diclofenac. The cytotoxicity effect, as assessed by the BSL assay, shows minimal toxicity at various concentrations, indicating the potential for further pharmaceutical applications.

The phytochemical analysis of various plant extracts, including *Diospyros montana* leaf extract, has shown that these extracts contain bioactive compounds such as flavonoids and phenolic compounds, which serve as effective reducing agents for synthesizing SeNPs. These SeNPs have demonstrated anti-bacterial activity against wound pathogens like *S. aureus*, *E. coli,* and *Aspergillus niger,* with zone of inhibition measurements indicating their potential therapeutic efficacy [24]. *Withania somnifera* leaf extract, another source of bioactive compounds, has also been used as an effective reducing agent for the synthesis of SeNPs. These SeNPs have shown anti-bacterial activity against *Bacillus subtilis*, *Klebsiella pneumoniae*, and *S. aureus*, further supporting the potential of plant-mediated SeNP synthesis for biomedical applications [25].

In summary, the study presents promising results on the synthesis of SeNPs using TP stem extract and their potential biomedical applications, including anti-bacterial, anti-oxidant, anti-inflammatory, and cytotoxicity effects. These findings suggest that TP stem-mediated SeNPs could be a valuable resource for developing new pharmaceutical products with therapeutic properties.

Limitations

The study on synthesizing SeNPs using TP stem extract presents promising biomedical applications but faces a few limitations. Primarily, it focuses on a narrow range of wound pathogens in vitro, lacks in vivo experiments, overlooks safety concerns, and biocompatibility. Further research should be conducted on more characterization techniques, in vivo studies, regulatory approvals, and long-term stability, facilitating the findings of TP stem-mediated SeNPs into practical biomedical applications.

## Conclusions

The current research explores the green synthesis method for the production of SeNPs from TP stem extract. The green-synthesized SeNPs were characterized using UV-vis spectrophotometer, FTIR, and SEM. The results show that the SeNPs have a great anti-microbial effect against wound pathogens. The anti-inflammatory and anti-oxidant activities show the highest inhibition percentage compared to the standard, in a concentration-dependent manner. Finally, the cytotoxic effect of synthesized SeNPs showed 70% live nauplii on day 2, proving it is less toxic. Further, in vivo studies are needed to explore the potential of green-synthesized SeNPs. In conclusion, the work lays the groundwork for future research into the biological uses of SeNPs synthesized using TP stem extract and provides valuable insights.
